# Improvement of Mesenchymal Stem Cell Immunomodulatory Properties by Heat-Killed *Propionibacterium acnes* via TLR2

**DOI:** 10.3389/fnmol.2018.00489

**Published:** 2019-01-10

**Authors:** Gabriela da Paz Silveira, Mayari Eika Ishimura, Daniela Teixeira, Layla Tesla Galindo, Agnes Araujo Sardinha, Marimelia Porcionatto, Ieda Maria Longo-Maugéri

**Affiliations:** ^1^Division of Immunology, Department of Microbiology, Immunology and Parasitology, Paulista School of Medicine, Federal University of São Paulo, São Paulo, Brazil; ^2^Division of Molecular Biology, Department of Biochemistry, Paulista School of Medicine, Federal University of São Paulo, São Paulo, Brazil

**Keywords:** *Propionibacterium acnes*, mesenchymal stem cell, immunomodulation, toll-like receptor 2, traumatic brain injury

## Abstract

Mesenchymal stem cells (MSCs) are an essential tool for regenerative medicine, which aims to develop new technologies to improve their effects to obtain useful transplantation results. MSC immunomodulatory role has been just demonstrated; however, how they react when they are stimulated by an adjuvant is poorly understood. Our group showed the adjuvant effect of killed *Propionibacterium acnes* (*P. acnes*) on hematopoietic stem cells. As these cells share the same MSCs bone marrow (BM) site and interact with each other, here we evaluated the *P. acnes* and its soluble polysaccharide (PS) effect on MSCs and their immunomodulatory role in a murine model of traumatic brain injury (TBI). The bacteria increased the absolute number of MSCs, including MSC subpopulations, and maintained MSC plasticity. *P. acnes* and PS enhanced MSC proliferation and improved their immunomodulatory effect. *P. acnes*-MSC and PS-MSC transplantation increased anti-inflammatory cytokine expression and diminished pro-inflammatory cytokine expression after injury. This effect seemed to be mediated via TLR2 since *P. acnes*-KOTLR2-MSC transplantation decreased TGF-β and IL-10 expression. Increasing in neural stem cells and neuroblasts after PS-MSC transplantation was also observed. The adjuvant effect of *P. acnes* is an alternative means of expanding MSCs and important to identify their subpopulations to know better their role under exogenous stimuli including inflammation resolution in an experimental model.

## Introduction

*Propionibacterium acnes (P. acnes)* is a gram-positive bacillus that belongs to normal human skin microbiota (Kligman et al., 1976; Webster, 1990; Leyden et al., 1998). In the past this bacteria was classified as *Corynebacterium parvum* and was employed in experimental and clinical studies as bacterial suspension killed by heating or phenol.

Heat-killed *P. acnes* has been used as a tool in studies of cellular immune responses due to its immunomodulatory effects (Green et al., 1977; Fisher et al., 1990). *P. acnes* increases macrophage phagocytic activity (Halpern et al., 1964) and resistance to several pathogens in mice (Nussenzweig, 1967; Brener and Cardoso, 1976; Teixeira et al., 1996; Mussalem et al., 2006); promotes Th1 and Th2 responses in mice (Braga et al., 2003; Squaiella et al., 2008); induces tumoricidal activity (Ananias et al., 2007); and has an essential role in the activation of various cell populations (Smith et al., 1993; Matsui et al., 1997; Yoneyama et al., 2001; Graham et al., 2004). The bacterial suspension is used after treatment by phenol or heat that preserve bacterial polysaccharides (PSs) and peptidoglycans. To identify the bacterial component responsible for the adjuvant effects our group phenol-extracted and purified a soluble PS from the *P. acnes* cell wall (Squaiella et al., 2006). This PS is involved in *P. acnes* adjuvant effects, such as increasing nitric oxide (NO) and TNF-α release, as well as the macrophages tumoricidal and phagocytic functions (Ananias et al., 2007) and also increases humoral and cellular immune response to *T. cruzi* DNA vaccine (Mussalem et al., 2006).

Squaiella et al. (2006) demonstrated that *P. acnes* and PS treatment enhances dendritic cell maturation (CD11c^+^ MHC II^+^) *in vivo* and *in vitro* as well as increases the number of bone marrow (BM) hematopoietic stem cells (HSCs). This effect could be correlated with the adjuvants’ ability to modulate different cell populations in blood, peripheral organs and tissues, such as peritoneal macrophages, NKT cells, and peritoneal and spleen B lymphocyte subpopulations (Kalis et al., 2005; Squaiella et al., 2006; Ananias et al., 2007; Mussalem et al., 2012). The effects of* P. acnes* could be partially explained by toll-like receptor (TLR) binding, mainly to TLR2, which is responsible for the recognition of peptidoglycans and lipoproteins (Takeda et al., 2003; Kalis et al., 2005).

The adjuvant effects on BM HSCs raise a question of whether *P. acnes* and PS also play a role in the activation of mesenchymal stem cells (MSCs). It was shown that MSCs are a critical component of the HSC niche and regulate its function (Calvi et al., 2003; Méndez-Ferrer et al., 2010). MSCs were first described by Friedenstein et al. (1968) as a multipotent population of non-hematopoietic cells in the BM. The International Society of Stem Cell Research, or ISSCR, established a minimum set of criteria for defining MSCs: plastic-adherent cells; capable of tri-lineage differentiation in bone, cartilage and fat; phenotypically positive for CD105, CD73 and CD90; and negative for CD45, CD34, CD11b, CD14, CD79a and HLA-DR. The expression level of these markers can vary based on culture conditions (English, 2013).

Several studies have demonstrated the regenerative potential of MSCs (Karussis et al., 2010), as these cells exhibit multipotent differentiation ability and can migrate and home into injury sites, where they are stimulated to secrete soluble factors that promote progenitor cell survival (Borlongan, 2011; Hong et al., 2011). This modulatory ability depends on the environment in which they are inserted. At injury sites, MSCs are activated by the presence of pro-inflammatory cytokines or TLR ligands. Some studies revealed that in the presence of TNF-α and ligands for TLR4 (lipopolysaccharide) or TLR2 (zymosan), MSCs were stimulated to produce cytokines responsible for the resolution of inflammation such as IL-10 (Nemeth et al., 2009; Choi et al., 2011).

BM-MSCs represent a small fraction of nucleated cells (approximately 0.001%–0.01%; Uccelli et al., 2006). The therapeutic application of MSCs often requires a large number of cells, and finding new approaches to increase MSC numbers and improve their modulatory properties are mandatory to upgrade clinical therapies.

Herein, we demonstrate the adjuvant effects of *P. acnes* and PS on MSC modulation *in vitro* and *in vivo*, including their impact in reducing the inflammatory response in a brain injury murine model. It is clear here that *P. acnes* as an adjuvant could be employed as an important tool to comprehend the different MSC functions, mainly about their subpopulations, under exogenous stimuli.

## Materials and Methods

### Animals

Eight- and Twelve-week-old C57BL/6j and TLR2 knockout female mice (KOTLR2) were used. Animals were kept in standard pathogen-free conditions on a 12-h light/dark cycle, at a controlled temperature, with water and food provided *ad libitum*. This study was carried out in strict accordance with the recommendations in the Guide for the Care and Use of Laboratory Animals of the Brazilian National Council of Animal Experimentation. The protocols were approved by the Ethics Committee on the Use of Laboratory Animals from Universidade Federal de São Paulo (CEUA 0244/12).

### Heat-Killed *P. acnes* Suspension (*P. acnes*) and Soluble Polysaccharide (PS) Extraction

*P. acnes* strain obtained from Adolfo Lutz Institute (São Paulo, Brazil) was cultured for 3 days at 37°C in anaerobic medium (Hemobac, Probac, São Paulo, Brazil). After washing by centrifugation three times at 2,000 *g* for 30 min, the bacterial pellet was resuspended in 0.9% saline and then autoclaved at 120°C for 20 min. The Bradford (1976) method was used to determine the protein concentration.

PS was obtained by phenol-extraction and ethanol precipitation, as previously described by our group (Squaiella et al., 2006) and based on Palmer and Gerlough’s (1940) protocol for PS extraction. Briefly, *P. acnes* was cultured, washed and autoclaved as previously described. To the volume of the bacteria suspension, an equal volume of 90% phenol was added. After 10 min at 70°C, the mixture was centrifuged at 2,000 *g* for 30 min. After this step, a density gradient was obtained, from which the aqueous phase and the PS ring were collected. This procedure was repeated twice. To the final volume collected, three volumes of absolute ethanol were added for PS precipitation. The solution was maintained overnight at 4°C and then centrifuged at 2,000 *g* for 30 min. After total evaporation of the residual ethanol, the precipitate was resuspended in 1 mL of distilled water and autoclaved. The carbohydrate concentration was determined by the Dubois method (Dubois et al., 1951), and the absence of protein was confirmed by the Bradford method (Bradford, 1976).

### Treatment Protocol

Wild-type (wt) mice received three subcutaneous injections, once per week, of heat-killed *P. acnes* suspension (140 μg of protein/350 μL) or 25 μg of PS in 350 μL. Control group was treated with 350 μL commercial 0.9% saline under the same conditions. Seven days after the last injection, blood and BM cells were obtained.

### MSCs Identification

Blood was collected by cardiac puncture of deeply anesthetized mice. Cells were obtained after the addition of 3 mL hemolytic buffer for 2 min followed by centrifugation at 400 *g* for 5 min. The cell pellet was suspended in 500 μL phosphate-buffered saline (PBS) and 2 mL hemolytic buffer for 2 min and then centrifuged at 400 *g* for 5 min.

To isolate MSCs from BM, epiphyses of femurs were cut, and the bone was flushed with low-glucose DMEM (Dulbecco’s Modified Eagle’s Medium, Gibco, San Francisco, CA, USA). The volume obtained was treated with hemolytic buffer for 2 min and centrifuged at 400 *g* for 5 min.

Cells from blood and BM were immunolabeled with the following monoclonal anti-mouse antibodies: CD3 (145-2C11), CD11b (M1/70), CD11c (N418), CD19 (1D3) and CD34 (RAM34), all conjugated with APC (eBioscience, San Diego, CA, USA), to define T lymphocytes, macrophages, dendritic cells, B cells and HSCs respectively. Cells negative for these markers were sorted (BD FACSAria™ II) and stained with CD73 PE-Cy7 (TY/11.8), CD90.2 FITC (Michel17) and CD105 PE (MJ7/18) anti-mouse antibodies (eBioscience) to define MSC subpopulations by flow cytometry (Attune^®^ Acoustic Focusing Flow Cytometer, Thermo-Fisher-Applied Biosystems, Waltham, MA, USA). The following subpopulations were characterized by single-, double- or triple-molecule expression as follows: CD90.2^+^, CD105^+^, CD73^+^, CD90.2^+^ CD105^+^, CD90.2^+^ CD73^+^, CD73^+^ CD105^+^ and CD90.2^+^ CD73^+^ CD105^+^. The absolute number of MSCs was calculated as the total number of cells (blood or BM) per animal times the percentage of sorted cells times the percentage of MSC positive markers (absolute number = total blood or BM cells/animal × %sorted cells × %MSC^+^).

### Purification of the Bone Marrow MSC Subpopulations From C57BL/6 Mice

BM cells obtained as described above were stained with monoclonal anti-mouse antibodies for CD3, CD11b, CD11c, CD19, and CD34 for negative selection and for CD73 (ecto-5′-nucleotidase), CD90.2 (Thy-1.2) and CD105 (endoglin; eBioscience, San Diego, CA, USA) for positive selection. The following subpopulations were sorted using a BD FACSAria™ II and used for transplantation assays: (a) CD73^+^; (b) CD90.2^+^; and (c) CD105^+^.

### Bone Marrow MSC Culture

BM cells obtained as described above from wt or KOTLR2 mice were suspended in low-glucose DMEM containing 10% fetal bovine serum (FBS, Cultilab, São Paulo, Brazil), 1% glutamine (Sigma Chemical Co., St. Louis, MO, USA), and 1% penicillin/streptomycin (Gibco). The total number of cells obtained per femur was cultured per well of 6-well plates (3 mL/well), at 5% CO_2_ and 37°C for 72 h. Cells that did not adhere were removed by changing the medium every 3 days until the adhered cells (MSCs) reached 70%–80% confluence. Cells were washed with 0.03% PBS/EDTA, detached with TrypLE™ Express (Gibco) for 5 min at 37°C. After washing with low-glucose DMEM containing 10% FBS for 5 min at 400 *g*, cells were recultured at 5% CO_2_ and 37°C in a 1:2 split ratio and the culture medium was replaced every 3 days. MSC culture was maintained until the third and fourth passages.

Cells cultured from wt mice were used in the differentiation potential, proliferation and transplantation experiments. Cells from KOTLR2 mice were used for transplantation only.

### MSC Multilineage Differentiation Potential

Differentiation potential was assessed by culturing MSCs from treated wt groups under conditions that induce adipogenic and osteogenic differentiation. Cells were seeded at a density of 1 × 10^4^ cells/well in a 24-well plate, and after 3 days, the medium was replaced with the adipogenic induction medium of the StemPro^®^ Adipogenesis Differentiation Kit (Gibco) or with the osteogenic induction medium of the StemPro^®^ Osteogenesis Differentiation Kit (Gibco). Cells were cultured for 21 days, and the medium was replaced three times per week. Adipogenesis was detected by Oil red O staining (Sigma), and osteogenesis detected by Alizarin Red S staining (Sigma). Staining was visualized using optical microscopy.

### Proliferation Assay

Third-passage MSCs from treated wt groups were collected and stained with 2 mL CFSE (1.25 μM) per 5 × 10^5^ cells (Cell Trace CFSE cell proliferation kit, Invitrogen, USA) for 15 min at 37°C. Cells were incubated in culture flasks and harvested at 3, 6 and 9 days. The mean fluorescence intensity (MFI) was measured using flow cytometry.

### Traumatic Brain Injury (TBI) Model

Twelve-week-old C57BL/6J mice were anesthetized with xylazine (32 mg/kg)/ketamine chlorhydrate (66 mg/kg; Dopalen, Vetbrands, São Paulo, Brazil) intraperitoneally. Animals were placed in a stereotactic frame, and a traumatic brain injury (TBI) was induced over the left motor cortex. A metal needle was frozen using isopentane on dry ice and then inserted four times for 30 s each into the motor cortex (stereotaxic coordinates from Bregma: AP +0.198 mm; ML +0.175 mm; DV −0.15 mm). Immediately after trauma, MSC subpopulations from the wt *P. acnes-*, PS- or saline-treated control groups sorted by FACSAria II, as described previously, or total MSCs of the third culture passage from treated wt or KOTLR2 groups were transplanted at the injury site. MSCs were suspended in low-glucose DMEM, and the cell concentration was adjusted to 1 × 10^5^ cells/3 μL. A control group received only vehicle (DMEM).

### Real Time Reverse Transcription Polymerase Chain Reaction (qPCR) to Quantify Cytokine Expression

Mice subjected to the TBI model were euthanized 24 h or 7 days after transplantation, and the left motor cortex was collected and stored at −80°C until analysis. Total RNA was obtained from the brain tissue specimens using Trizol reagent (Invitrogen). Reverse transcriptase reactions were performed with the ImProm-II Reverse Transcription System (Promega, Madison, WI, USA) using 2 μg total RNA. qPCR of the brain tissue samples was performed using a PRISM 7500 Sequence Detector, Sequence Detection Software 1.9 for analysis (Applied Biosystems), and SYBR Green PCR Master Mix (Applied Biosystems). Expression of the following genes was analyzed: *IL-4, IL-6, IL-10, TGF-β* and *TNF-α*. *HPRT* and *GAPDH* were used as reference genes, and relative gene expression levels were determined according to the manufacturer’s ΔΔCt method (Applied Biosystems). Values are expressed relative to control RNA obtained from motor cortex samples collected from mice that received low-glucose DMEM (vehicle) or from MSCs collected from the saline group (MSCs). The primers used are described in the Supplementary Table S1.

### Immunohistochemistry

Two hours after TBI and MSC transplantation, the mice were injected intraperitoneally with 5-Bromo-2′-deoxyuridine (BrdU; Sigma; 75 mg/kg). After 24 h or 7 days, the mice were transcardially perfused. Mice in the 7-day group received one dose of BrdU/day. They were anesthetized, and transcardiac perfusion was performed with 4% paraformaldehyde. The brain was then dehydrated with 30% sucrose for 48 h. Brain coronal sections (20 μm) were incubated overnight with anti-GFAP (Millipore, Temecula, CA, USA), anti-DCX (Millipore), and anti-BrdU (Axyll, New York, NY, USA) primary antibodies at 4°C. Then, the slices were incubated at 37°C for 1 h with secondary antibodies labeled with Alexa Fluor^®^ 488 or Alexa Fluor^®^ 594 (Invitrogen). Images were acquired using scanning fluorescence microscopy (Leica Microsystems, Wetzlar, Germany). For statistical analysis, we selected three slices from each brain for staining (*n* = 3 for each group).

### Statistical Analysis

Statistically significant differences between control and treated groups were evaluated by one-way analysis of variance (ANOVA) followed by Tukey’s post-test, and between groups and different days post-treatment were evaluated by two-way ANOVA followed by Bonferroni’s post-test, using GraphPad Prism software version 5.01 (GraphPad Software, USA).

## Results

### *P. acnes* and PS Elevated the Absolute Number of MSCs in the Blood and Bone Marrow

After negative selection, MSCs were sorted as positive for CD73, CD90.2 and CD105 (Supplementary Figure S1). The blood and BM MSCs single-positive for CD73 (*F*_(2,6)_ = 13.48, *p* = 0.006; and *F*_(2,6)_ = 8.32, *p* = 0.019), CD90.2 (*F*_(2,6)_ = 11.86, *p* = 0.008; and *F*_(2,6)_ = 23.83, *p* = 0.001) and CD105 (*F*_(2,6)_ = 8.26, *p* = 0.019; and *F*_(2,6)_ = 11.75, *p* = 0.008) were increased after treatment with *P. acnes* and PS (*p* < 0.05).

**Figure 1:**
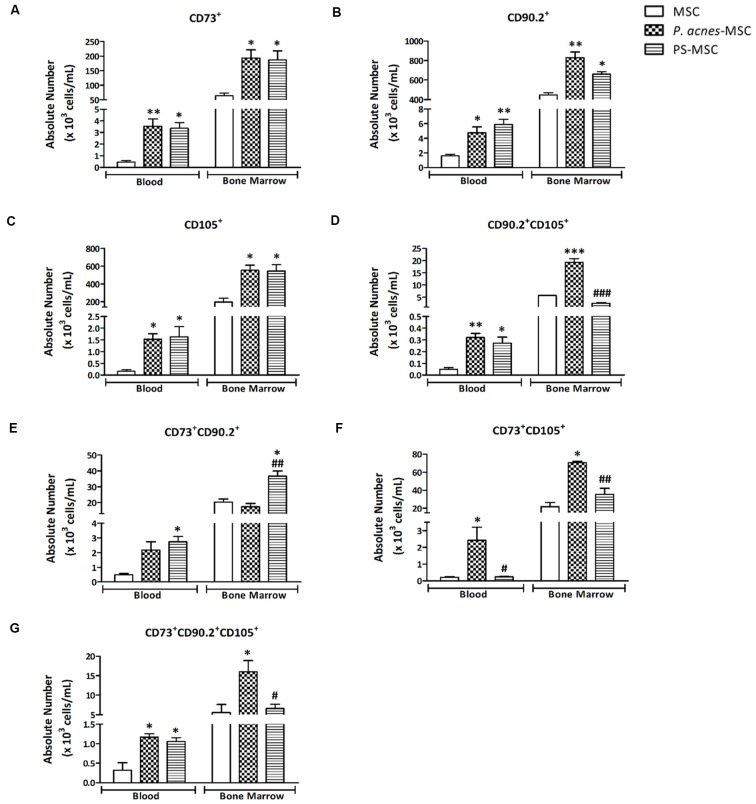
*P. acnes* and polysaccharide (PS) treatments enhance specific subpopulations of blood and bone marrow (BM) mesenchymal stem cells (MSCs). One week after mice were treated with *P. acnes*, PS or saline, MSCs were isolated from blood and BM, immunolabeled and sorted by negative expression of CD3, CD11b, CD11c, CD19, and CD34. MSCs were characterized and quantified using the positive expression for CD73, CD90.2 and CD105 markers. The following MSC subpopulations were identified: **(A)** CD73^+^, **(B)** CD90.2^+^, **(C)** CD105^+^, **(D)** CD90.2^+^ CD105^+^, **(E)** CD73^+^ CD90.2^+^, **(F)** CD73^+^ CD105^+^, and **(G)** CD73^+^ CD90.2^+^ CD105^+^. Data are presented as mean ± SEM of three mice per group, from a representative experiment of three independent experiments. *p < 0.05, **p < 0.001 and ***p < 0.0001, when significance was calculated in relation to the MSC group, and ^#^*p* < 0.05, ^##^*p* < 0.001 and ^###^*p* < 0.0001, when significance was calculated in relation to the *P. acnes*-MSC group, determined by one-way analysis of variance (ANOVA) followed by Tukey’s post-test.

In the blood*, P. acnes* treatment increased the CD73^+^ MSCs (3.5 × 10^3^ cells/mL), CD90.2^+^ MSCs (4.7 × 10^3^ cells/mL) and CD105^+^ MSCs (1.5 × 10^3^ cells/mL) and the PS treatment elevated the CD73^+^ MSCs (3.3 × 10^3^ cells/mL) CD90.2^+^ MSCs (5.9 × 10^3^ cells/mL) and CD105^+^ MSCs (1.6 × 10^3^ cells/mL) when compared to the respective controls (0.5 × 10^3^ cells/mL; 1.6 × 10^3^ cells/mL; 0.16 × 10^3^ cells/mL; Figure 1).

The CD73^+^ BM-MSCs was augmented after *P. acnes* (1.9 × 10^5^ cells/mL) and PS treatment (1.8 × 10^5^ cells/mL) when compared to control (0.64 × 10^5^ cells/mL; Figure 1A). The CD90.2^+^ BM-MSCs and CD105^+^ BM-MSCs respectively (Figures 1B,C) in the *P. acnes* (8.3 × 10^4^ cells/mL, 5.5 × 10^5^ cells/mL) and PS (6.6 × 10^4^ cells/mL, 5.4 × 10^5^ cells/mL) groups was also increased relative to the control group (4.5 × 10^4^ cells/mL, 2 × 10^5^ cells/mL).

When we evaluated subpopulations that expressed two or three markers, the results were similar; however, they depended on location (blood or BM). *P. acnes* treatment increased the CD90.2^+^ CD105^+^ BM-MSCs (1.9 × 10^4^ cells/mL; *F*_(2,6)_ = 109.1, *p* < 0.0001) and also in the blood (3.2 × 10^2^ cells/mL; *F*_(2,6)_ = 15.91, *p* = 0.004) while PS elevated this subtype only in the blood (2.7 × 10^2^ cells/mL) relative to the respective control groups (BM-MSCs 0.57 × 10^4^ cells/mL and Blood MSCs 0.4 × 10^2^ cells/mL; Figure 1D).

The absolute number of CD73^+^ CD90.2^+^ BM-MSCs in the PS group (3.6 × 10^4^ cells/mL) was increased (*F*_(2,6)_ = 16.24, *p* = 0.0038) compared with that in the control (2 × 10^4^ cells/mL) and *P. acnes* (1.7 × 10^4^ cells/mL) groups (Figure 1E). In blood, this subset remained significantly increased (*F*_(2,6)_ = 8.57, *p* = 0.0175) after PS treatment (2.7 × 10^3^ cells/mL).

The CD73^+^ CD105^+^ BM-MSCs was significantly increased (*F*_(2,6)_ = 26.40, *p* = 0.0011) after both *P. acnes* (7 × 10^4^ cells/mL) and PS (3.5 × 10^4^ cells/mL) treatment compared to the control group (2.1 × 10^4^ cells/mL; Figure 1F). The same subset in blood was significantly increased (*F*_(2,6)_ = 7.74, *p* = 0.0218) after *P. acnes* treatment (2.4 × 10^3^ cells/mL) compared to control and PS groups.

When we evaluated BM-MSCs CD90^+^ CD105^+^ CD73^+^, only *P. acnes* increased (*F*_(2,6)_ = 7.21, *p* = 0.0254) the absolute number (1.6 × 10^4^ cells/mL) when compared to control (0.5 × 10^4^ cells/mL; Figure 1G). In the blood, both *P. acnes* (1.1 × 10^2^ cells/mL) and PS (1 × 10^2^ cells/mL) treatment significantly increased (*F*_(2,6)_ = 11.56, *p* = 0.0088) this subpopulation relative to the control (0.3 × 10^2^ cells/mL).

### *P. acnes* and PS Induced MSC Proliferation *in vitro*

**Figure 2:**
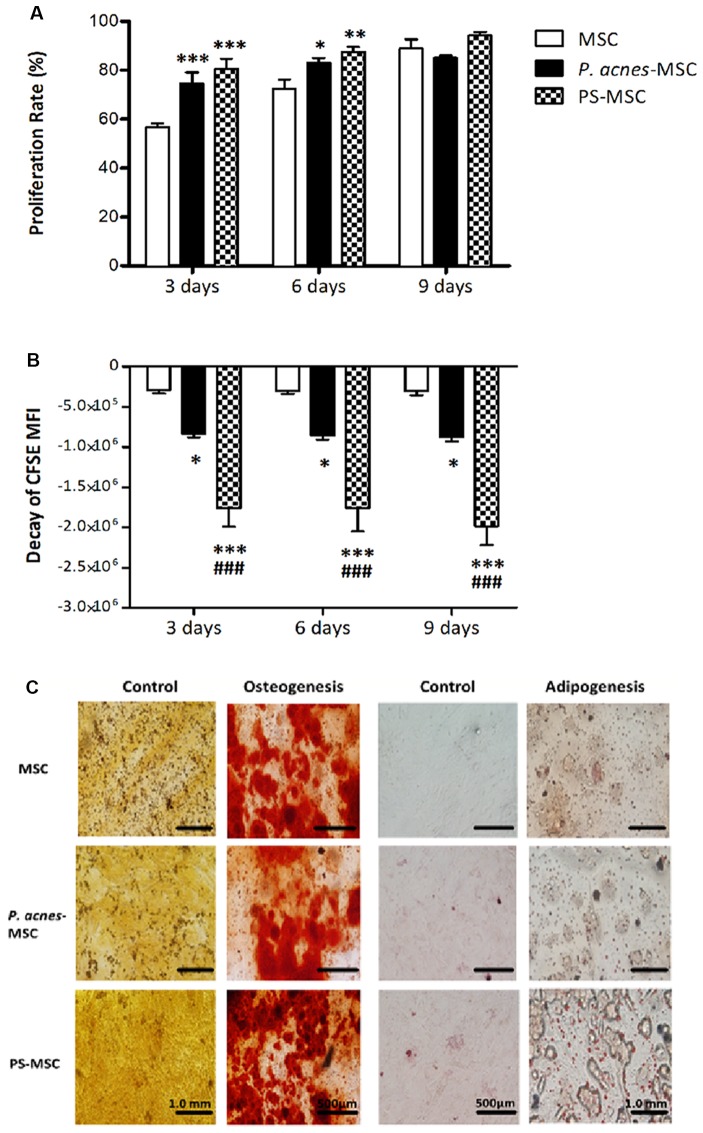
*P. acnes* and PS treatment increase MSC proliferation without affecting differentiation. Cells obtained from *P. acnes*-, PS- and saline-treated groups were stained with CFSE and cultured for 9 days. **(A)** MSC proliferation rate analysis at 3, 6 and 9 days. **(B)** The decay of mean fluorescence intensity (MFI) proliferative cycles after 3, 6 and 9 days of culture. **(C)** MSCs were cultured under control, osteogenic or adipogenic conditions for 21 days. Osteogenic differentiation was observed after Alizarin Red S staining, and calcified nodules can be seen in red in all groups. Adipogenic differentiation was observed after Oil Red O staining, and lipid vesicles appear in red. Data are presented as the mean ± SEM of three mice per group, from two independent experiments. *p < 0.05, **p < 0.001 and ***p < 0.0001, when significance was calculated in relation to the MSC group, and ^###^*p* < 0.0001, when significance was calculated in relation to the *P. acnes*-MSC group, determined by two-way ANOVA followed by Bonferroni’s post-test.

MSCs obtained from wt mice treated groups displayed higher proliferation rates than those from the control group (Figure 2A, *F*_(2,42)_ = 16.88, *p* < 0.0001, and Figure 2B, *F*_(2,42)_ = 87.24, *p* < 0.0001). On the third day, the proliferation rate of MSCs from the control group was 56.6%, while that of MSCs from the *P. acnes-* and PS-treated groups was 74.5% and 80.5%, respectively. The increase was 17.9% (*P. acnes*) and 23.9% (PS) relative to the control. After 6 days, the proliferation rate of the MSCs remained elevated at 83.1% and 87.6% (*P. acnes* and PS, respectively) relative to 72.4% in control, corresponding to an increase in MSC proliferation of 10.7%–15.2%. After 9 days, there was no significant difference between the groups (Figure 2A).

However, when we analyzed MFI decay, we observed a more significant decrease at all evaluation time points, including the 9th day after culture, for MSCs from *P. acnes*- and PS-treated mice (Figure 2B). The MFI of CFSE-stained MSCs from the *P. acnes*-treated group was decreased by approximately 2.8 times relative to that of the control MSCs in all periods analyzed. Relative to the control MSCs, MSCs from the PS-treated group displayed a 6-fold reduction after 3 and 6 days and a 6.5-fold decrease after 9 days. These results indicate that cells from the adjuvant-treated groups completed more proliferative cycles than cells from the control group.

### *In vitro* MSC Plasticity

MSCs from BM from *P. acnes*-treated group (*P. acnes*-MSCs), PS-treated group (PS-MSCs) and saline control group (MSCs) adhered to plastic surfaces and were capable of differentiating into adipocytes and osteocytes. Osteocyte differentiation was identified by the deposition of calcium (red), and adipocyte differentiation was verified by lipid accumulation (red; Figure 2C), indicating that the adjuvants did not alter MSC plasticity.

### Improvement of MSC Immunomodulatory Properties by *P. acnes* and PS

**Figure 3:**
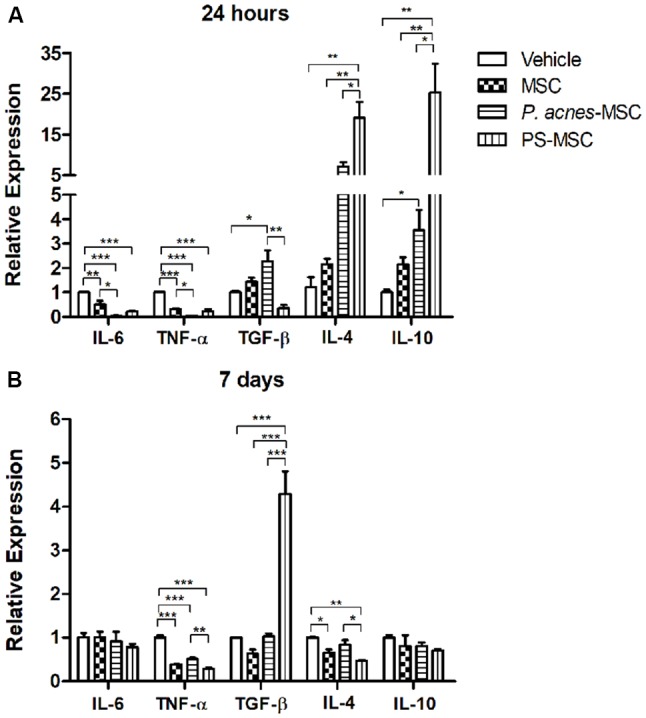
Transplantation of MSCs isolated from *P. acnes*- and PS-treated mice at an injury site in the cerebral cortex alters cytokine expression. RNA was extracted from motor cortex **(A)** 24 h and **(B)** 7 days after traumatic brain injury (TBI) and MSC transplantation. Quantitative polymerase chain reaction (qPCR) was performed, and cytokine expression was calculated relative to HPRT expression. Data are expressed as the mean (using the 2^−ΔΔCt^ method) ± SEM of 3 mice per group, from a representative experiment of three independent experiments. *p < 0.05, **p < 0.001 and ***p < 0.0001, determined by one-way ANOVA followed by Tukey’s post-test.

Twenty-four hours after cell transplantation, cytokine expression was analyzed. *P. acnes-*MSCs promoted a significant reduction in pro-inflammatory cytokine expression compared to saline–MSCs. IL-6 expression was decreased (*F*_(3,8)_ = 28.02, *p* = 0.0001) by 14-fold relative to that in the MSC group and 29-fold relative to that in the vehicle group (Figure 3A). TNF-α expression was reduced (*F*_(3,8)_ = 58.78, *p* < 0.0001) by 14.5-fold relative to that in the control MSC group and 50-fold relative to that in the vehicle group. Concerning anti-inflammatory cytokines, *P. acnes*-MSCs increased TGF-β (2.2-fold, *F*_(3,8)_ = 10.33, *p* = 0.004), IL-4 (7.1-fold* F*_(3,8)_ = 15.82, *p* = 0.001), and IL-10 (2.3-fold, *F*_(3,8)_ = 15.82, *p* = 0.001) expression relative to vehicle. IL-4 expression was 3.3-fold higher than that in the MSC group. Likewise, PS-MSCs increased the expression of IL-4 and IL-10 by 19- and 25.3-fold, respectively, relative to control MSCs. TGF-β expression was decreased by approximately 2.8-fold after PS-MSC transplantation comparable to that in the vehicle group and by 4.1-fold relative to that in the MSC group. PS-MSCs also reduced IL-6 and TNF-α expression compared to vehicle.

Seven days after injury, the down-regulation of TNF-α persisted in all groups (*F*_(3,8)_ = 90.23, *p* < 0.0001): 2-fold for* P. acnes-*MSCs, 3.4-fold for PS-MSCs and 2.5-fold for MSCs compared to vehicle (Figure 3B and Supplementary Figure S2). PS-MSCs enhanced TGF-β expression by 6.8-fold relative to MSCs and by 4.2-fold compared to vehicle (*F*_(3,8)_ = 42.80, *p* < 0.0001). IL-4 expression was reduced (*F*_(3,8)_ = 11.48, *p* = 0.0029) by 1.5-fold in the MSC group and by 2.1-fold in the PS-MSC group relative to that in the vehicle group.

### Improvement of the Immunomodulatory Properties of MSC Subpopulations by *P. acnes* and PS Treatment

**Figure 4:**
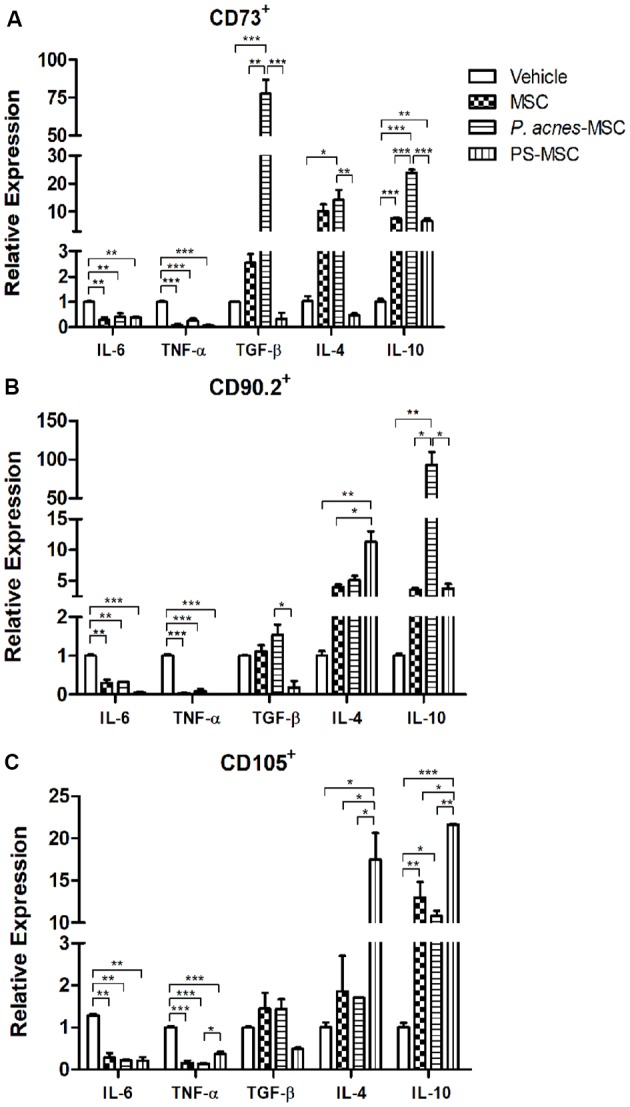
Transplantation of MSC subpopulations isolated from *P. acnes*- and PS-treated mice at an injury site in the cerebral cortex alters cytokine expression. Twenty-four hours after TBI and MSC subpopulation transplantation (**A**, CD73^+^ MSC; **B**, CD90.2^+^ MSC; and **C**, CD105^+^ MSC), RNA was extracted from the motor cortex. qPCR was performed, and cytokine expression was calculated relative to *HPRT* expression. Data are expressed as the mean (using the 2^−ΔΔCt^ method) ± SEM of three mice per group, from a representative experiment of three independent experiments. *p < 0.05, **p < 0.001 and ***p < 0.0001, determined by one-way ANOVA followed by Tukey’s post-test.

Compared to vehicle, CD73^+^, CD90.2^+^ and CD105^+^ MSC subpopulations from all treatment groups (Figure 4) could decrease IL-6 (*F*_(3,8)_ = 14.02, *p* = 0.0015; *F*_(3,4)_ = 100.8, *p* = 0.0003; and *F*_(3,4)_ = 58.45, *p* = 0.0009) and TNF-α (*F*_(3,8)_ = 70.09, *p* < 0.0001; *F*_(3,4)_ = 201.1, *p* < 0.0001; and *F*_(3,8)_ = 86.62, *p* < 0.0001) expression 24 h after implantation into the injury site.

*P. acnes*-MSC-CD73^+^ treatment (Figure 4A) increased the expression of all anti-inflammatory cytokines (TGF-β, *F*_(3,4)_ = 70.58, *p* = 0.0006; IL-4, *F*_(3,8)_ = 9.54, *p* = 0.0051; and IL-10, *F*_(3,8)_ = 183.7, *p* < 0.0001) relative to vehicle treatment. Moreover, *P. acnes*-MSC-CD73^+^ caused a 35-fold increase in TGF-β expression and a 3-fold increase in IL-10 expression compared to the corresponding values in the MSC-CD73^+^ group. On the other hand, PS-MSC-CD73^+^ treatment enhanced IL-10 expression only relative to vehicle and reduced TGF-β (7.3-fold) and IL-4 expression (20.8-fold) relative to MSC-CD73^+^ treatment.

Treatment with *P. acnes* and PS upregulated the expression of IL-4 (*F*_(3,4)_ = 20.72, *p* = 0.0067) and IL-10 (*F*_(3,4)_ = 28.39, *p* = 0.0037) induced by CD90.2^+^ cells (Figure 4B) compared to vehicle. PS-MSC-CD90.2^+^ transplantation, compared to MSC-CD90.2^+^ transplantation, enhanced IL-4 expression by 2.8-fold. *P. acnes*-MSC-CD90.2^+^ transplantation caused the most pronounced increase in IL-10 expression, which was approximately 26-fold relative to that in the MSC-CD90.2^+^ group. TGF-β expression was reduced only in the PS-MSC-CD90.2^+^ group compared to that in the MSC-CD90.2^+^ (6-fold) and vehicle (5.5-fold) groups.

PS-MSC-CD105^+^ transplantation (Figure 4C) increased IL-4 (9.5-fold, *F*_(3,4)_ = 24.72, *p* = 0.0048) and IL-10 expression (1.6-fold, *F*_(3,4)_ = 75.96, *p* = 0.0006) relative to MSC-CD105^+^ transplantation. Besides, compared to vehicle, PS-MSC-CD105+ treatment increased IL-4 expression by 17.5-fold and IL-10 expression by approximately 21.6-fold. *P. acnes*-MSC-CD105^+^ treatment enhanced IL-10 expression only compared to vehicle; this increase was approximately 10.8-fold. No differences relative to MSC-CD105^+^ treatment were observed (Figure 4C and Supplementary Figure S3).

### TLR2 Activation Is One of the Mechanisms by Which *P. acnes* Modulates the Effects of MSCs on Cytokine Expression

**Figure 5:**
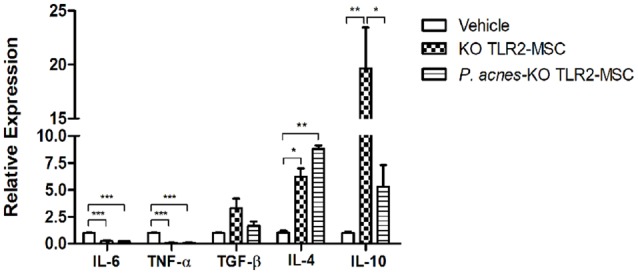
Transplantation of KOTLR2-MSCs isolated from *P. acnes*- and PS-treated mice at an injury site in the cerebral cortex alters cytokine expression. Twenty-four hours after TBI and TLR2 knock out MSC transplantation in wild-type (wt) mice, RNA was extracted from the motor cortex. qPCR was performed, and cytokine expression was calculated relative to *HPRT* expression. Data are expressed as the mean (using the 2^−ΔΔCt^ method) ± SEM of three mice per group, from a representative experiment of three independent experiments. *p < 0.05, **p < 0.001 and ***p < 0.0001, determined by one-way ANOVA followed by Tukey’s post-test.

We analyzed the effects of transplantation of MSCs isolated from KOTLR2 mice treated with *P. acnes* or saline in a brain injury model. The absence of TLR2 abolished the effect of *P. acnes* on the relative expression of IL-4 (*F*_(2,6)_ = 14.8, *p* = 0.0048) and TGF-β (*F*_(2,6)_ = 4.34, *p* = 0.0682), which are both anti-inflammatory cytokines, as the results using MSCs from KOTLR2 mice were similar to those obtained when untreated KOTLR2-MSCs were transplanted (Figure 5). Besides, the lack of TLR2 strongly diminished the effect of KOTLR2-MSCs from the *P. acnes* group on IL-10 expression (*F*_(2,6)_ = 15.62, *p* = 0.0042), as the IL-10 expression was higher when KOTLR2-MSCs from the saline group were used (Figure 5 and Supplementary Figure S4). It seems clear that most of the effects induced by *P. acnes* depend on the TLR2 pathway.

### PS-MSC Transplantation Stimulated Neurogenesis After Brain Injury

**Figure 6:**
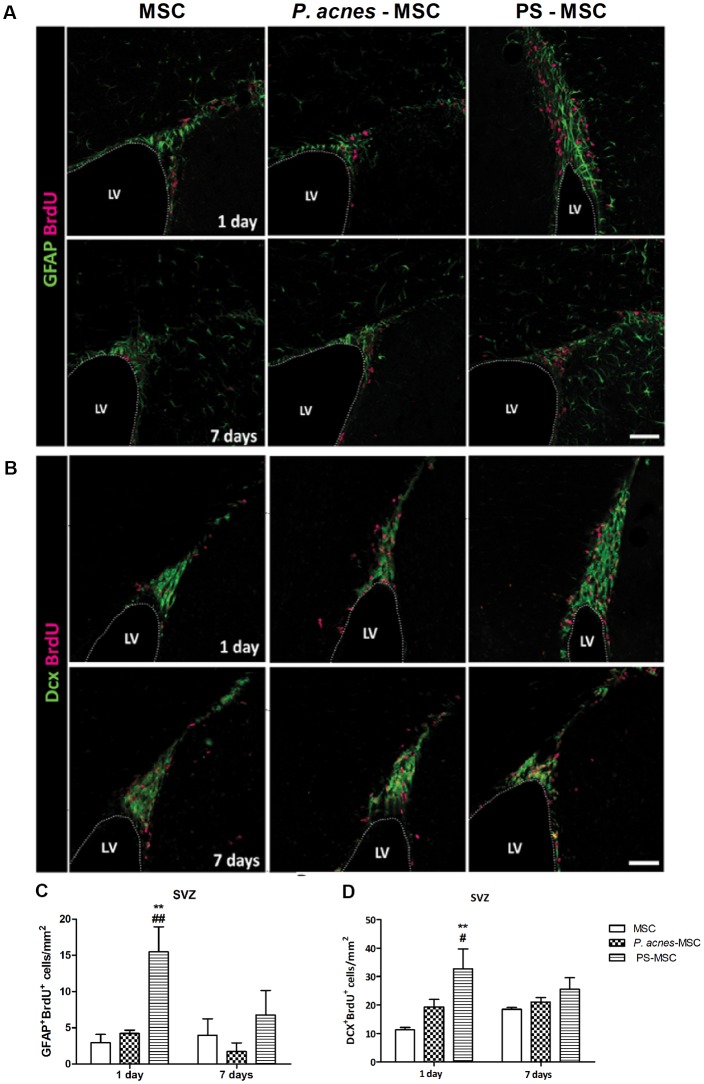
MSCs isolated from PS-treated mice transplanted at an injury site in the cerebral cortex increase neural stem cell proliferation. **(A)** Immunostaining for GFAP (green) and BrdU (red) in the SVZ, 1 and 7 days after TBI. **(B)** Immunostaining for DCX (green) and BrdU (red) in the SVZ, 1 and 7 days after TBI. **(C)** Quantification of GFAP^+^BrdU^+^ cells in the SVZ. **(D)** Quantification of DCX^+^BrdU^+^ cells in the SVZ. Scale bar: 50 μm. Data are presented as mean ± SEM of three mice per group, from a representative experiment of three independent experiments. **p < 0.001, when significance was calculated in relation to the MSC group, and ^#^*p* < 0.05 and ^##^*p* < 0.001, when significance was calculated in relation to the *P. acnes*-MSC group, determined by two-way ANOVA followed by Tukey’s post-test. BrdU, 5-Bromo-2’-deoxyuridine; DCX, doublecortin; GFAP, glial fibrillary acidic protein; LV, lateral ventricle; SVZ, subventricular zone.

Quantification of GFAP^+^BrdU^+^ (neural stem cells; Figures 6A,C, *F*_(2,12)_ = 7.94, *p* = 0.0064) and DCX^+^BrdU^+^ (neuroblasts; Figures 6B,D, *F*_(2,12)_ = 8.18, *p* = 0.0057) cells in the subventricular zone (SVZ) 24 h after injury revealed an increase in the numbers of both cells, by approximately 5.3- and 2.9-fold, respectively, relative to the control, after MSC-PS transplantation. The numbers of neural stem cells were also enhanced by 3.6 times compared to the numbers in the MSC group. After 7 days, there were no differences among the groups.

## Discussion

Herein we demonstrated the adjuvant effect of a heat-killed *P. acnes* suspension on the expression of chemokines by MSCs. We show here that the PS of *P. acnes* mediates the modulatory effects observed for these cell populations.

The results revealed that *P. acnes* and PS act as important tools to hasten the acquisition of BM-MSC-enriched cultures since treated cells have better proliferation rates, complete more proliferative cycles (Figures 1A,B), without, however, losing their ability to differentiate, as qualitatively demonstrated by the present osteogenic and adipogenic differentiations (Figure 1C). In addition to improving proliferation, *P. acnes* promotes the enhancement of MSC immunomodulatory properties. In this study, we chose a TBI animal model to evaluate cerebral inflammatory responses to *P. acnes*-MSC transplantation. TBI triggers an inflammatory process that acts in neurogenic areas, changing the microenvironment of neural stem cells as well as their proliferation, migration and differentiation processes (Peng et al., 2015). Microglia and perivascular macrophages with a classical inflammatory profile contribute to this process, and an uncontrolled immune response impairs neural progenitor cell growth and reparatory processes (Nemeth et al., 2009). However, microglia aid in recovery from injury when they acquire an anti-inflammatory profile (Shechter and Schwartz, 2013). In previous studies, MSCs were used as a strategy to change the microglial profile and counteract the pathology induced by trauma (Zanier et al., 2014; Gao et al., 2016). MSC transplantation stimulates injury repair after local or systemic administration by reducing the expression of pro-inflammatory cytokines such as IL-1β, IL-6, TNF-α, INF-γ as well as by enhancing the expression of the anti-inflammatory factors TGF-β and IL-10, which are related to tissue repair (Mahmood et al., 2004; Liu et al., 2009; Galindo et al., 2011).

Compared to MSCs, *P. acnes*-MSCs promoted a more significant decrease in the expression of IL-6 and TNF-α at the injury site at 24 h after trauma. This finding reveals a significant adjuvant modulatory effect since these cytokines are responsible for glial cell activation, which is related to increased tissue damage during the acute phase of injury. A high TNF-α concentration causes neuronal apoptosis by extrinsic pathway activation and receptor depletion reduced neuronal damage (McCoy and Tansey, 2008). Seven days after transplantation of MSCs derived from all groups (untreated, *P. acnes* and PS), TNF-α expression remained decreased, reinforcing a neuroprotective role for MSCs. However, we did not detect a difference in IL-6 expression among the groups after 7 days (Figure 3). The role of IL-6 in the chronic lesion seems to be essential to the revascularization process (Morganti-Kossmann et al., 2007; Suzuki et al., 2009; Galindo et al., 2011); MSCs could modulate this mechanism to repair lesioned tissue.

We could observe that both *P. acnes*-MSC relative to the vehicle in the acute phase and PS-MSC in chronic phase (7 days) promoted an increase in TGF-β expression in the site of the lesion. The long-term presence of TGF-β after injury is associated with clinical improvement (Ma et al., 2008). TGF-β promotes neuronal differentiation and survival after a stroke and prevents the loss of neural cells and consequent functional harm in a TBI model (Wang et al., 2015). Increasing of TGF-β expression by *P. acnes* was described since 1995 by Masuhara (1995) that demonstrated elevated levels in the liver of injected rats after *P. acnes* treatment.

The increase in IL-4 expression that occurred 24 h after transplantation of *P. acnes*-MSCs and PS-MSCs is consistent with the decrease in the expression of pro-inflammatory cytokines. This is because IL-4 can inhibit the IL-6 and TNF-α synthesis polarizing to Th2 response (Xiong et al., 2011). In other models of brain injury, IL-4 has been implicated in the control of the inflammatory response and consequently, in animal recovery (Yang et al., 2016). IL-10 is another critical cytokine for the outcome of neuroinflammation. We demonstrated that PS-MSC elevated the IL-10 expression in the site of the lesion when compared to MSCs. This cytokine is related to the suppression of glial cell activity, maintenance of the anti-apoptotic protein Bcl-2, enhancement of neurogenesis and reduction in the size of the injured area (Liu et al., 2007; Du et al., 2008).

*P. acnes*-mediated stimulation of MSCs promotes a favorable environment and intensifies the production of reparative factors, which leads to the proliferation of endogenous stem cells that can act in tissue repair. In the brains of adult rodents, the SVZ of the lateral ventricles (LVs) and the subgranular zone of the hippocampal dentate gyrus are neurogenic niches where neural stem cells are generated. In the SVZ, there are four cell types: (1) ependymal cells lining the LV; (2) astrocyte-like cells, which are considered true neural stem cells with the potential for self-renewal; (3) transient amplifying cells, which originate from neural stem cells; and (4) migratory neuroblasts, which are cells that reach their final location in the central nervous system after traveling a path signaled by soluble factors and components of the extracellular matrix (Alvarez-Buylla and Lim, 2004; Kaneko and Sawamoto, 2009; Nam et al., 2009).

Stem cells present in the SVZ niche are induced to proliferate and give to neural precursors that home to injury sites in the central nervous system under the direction of locally secreted chemokines (Dixon et al., 1997; Martino and Pluchino, 2006). An increase of neural stem cells proliferation was observed when they are cultivated with MSCs soluble factors (Galindo et al., 2011). Here we found that PS-MSCs could enhance the number of neural stem cells (GFAP^+^BrdU^+^) in the SVZ and the number of newly generated neuroblasts (DCX^+^BrdU^+^). This effect seems due to the increase in IL-4 and IL-10, which stimulate the synthesis of trophic factors such as brain-derived neurotrophic factor (BDNF). BDNF improves locomotor function in spinal cord injury models and is also an important survival factor for neurons (Snapyan et al., 2009).

MSC culture is heterogeneous containing subpopulations at different stages of differentiation that can result in distinct effects when they are transplanted (Tremain et al., 2001; Sempere et al., 2014). To obtain more promising therapeutic results, efforts are being made to identify subpopulations of MSCs that can respond more adequately to the microenvironment in which they are transplanted. First, we evaluated which subpopulations of MSCs *P. acnes* were able to stimulate in blood and BM. Our results revealed a significant increase in the absolute numbers of blood and BM MSC subpopulations that are CD90.2^+^, CD73^+^, CD105^+^, CD90.2^+^ CD105^+^, CD73^+^ CD105^+^ and CD90.2^+^ CD105^+^ CD73^+^ after *P. acnes* treatment. PS enhanced CD90.2^+^ CD105^+^ and CD90.2^+^ CD105^+^ CD73^+^ MSC subpopulations in the blood as well as CD90.2^+^ CD73^+^ BM-MSCs. The differences observed in the subpopulations may be associated with differences in the stage of cell maturation. Furthermore, the cells may or may not express receptors that enable their interaction with the adjuvant, such as TLR2, or chemokine receptors related to MSC mobilization into the injury site, such as CCL2 and CXCL12 (Belema-Bedada et al., 2008). It has been demonstrated that* P. acnes* increases chemokines synthesis as CCL2 and IL-8 in different cell types (Ichiyasu et al., 1999; Nagy et al., 2005).

Every subpopulation analyzed was stimulated by the adjuvants, so we chose to evaluate the immunomodulatory function of three major MSC subtypes, namely, CD73^+^, CD90.2^+^ and CD105^+^ MSCs, 24 h after the induction of a TBI model. CD73^+^, CD90.2^+^ and CD105^+^ MSCs isolated from mice subjected to both treatments maintained their modulatory effects of decreasing IL-6 and TNF-α expression at the injury site. TGF-β seems to be influenced by the presence of CD73^+^ cells since only this subpopulation stimulated TGF-β expression. Furthermore, the most pronounced enhancement of TGF-β expression was observed in the *P. acnes*-MSC-CD73^+^ group, showing an adjuvant effect of *P. acnes* specifically on the CD73^+^ subtype. The correlation between CD73 (ectonucleotidase) molecule and TGF-β can be partially explained by the influence of TGF-β in reducing the expression of ectonucleotidase repressors, such as growth factor independent of protein-1 (Gfi-1; Regateiro et al., 2011; Chen et al., 2016). On the other hand, PS down-regulated this cytokine as well as IL-4 expression, suggesting that other *P. acnes* compounds are responsible for that modulation.

The influence of the adjuvants on the CD90.2^+^ cells was evident based on the pronounced enhancement of IL-4 expression in the PS group and of IL-10 expression in the *P. acnes* group relative to the effects of MSCs from untreated mice. The CD90.2 molecule may be associated with MSC immunosuppressive abilities because low CD90.2 expression decreases IL-10 production (Campioni et al., 2009). Several studies have clearly demonstrated effects of *P. acnes* on the expression of surface molecules associated with cell maturation, antigen presentation (CD80, CD86 and CD28; MacDonald et al., 2001; Squaiella et al., 2006) and cell migration in macrophages (MCP-1; Ichiyasu et al., 1999). MSCs expressing CD105 have immunomodulatory properties since they induce an anti-inflammatory profile in macrophages, an effect that is associated with IL-10 (Ishimura et al., 2008; Anderson et al., 2013). Our results showed that PS-MSC-CD105^+^ treatment enhanced IL-4 and IL-10 expression.

*P. acnes* was described as a typical Th1-inducer antigen, associated to increase of proinflammatory cytokines. However, our group demonstrated that *P. acnes* was able to potentiate the Th2 to ovalbumin or change it to a Th1 response in a murine model of type I hypersensitivity reaction (Braga et al., 2003; Squaiella et al., 2008). This Th1/Th2 polarization effect corroborates our findings regarding the adjuvant-mediated improvement of MSC properties. The mechanism by which *P. acnes* and PS modulate MSCs is unclear; however, we believe the majority of the effects may occur via TLR2 since MSCs express TLR (DelaRosa and Lombardo, 2010) and because our group demonstrated that *P. acnes* increases the expression of TLR2, TLR4, TLR9 in macrophages, dendritic cells, B and B-1 lymphocytes (Kalis et al., 2005; Mussalem et al., 2012; Gambero et al., 2016). Previously, we demonstrated that B-1 cells from KOTLR2 mice lost their differentiation to phagocytes ability when animals were treated with *P. acnes* (Gambero et al., 2016). Besides, the treatment of mice with *P. acnes* increases the expression of TLR2, suggesting the same effect as the agonist since the cells become activated and induce the expression of several surface molecules such as CD80, CD86 chemokine receptor and cytokines (Squaiella et al., 2006, 2008; Mussalem et al., 2012; Squaiella-Baptistão et al., 2015). It was also observed by Kim et al. (2002) that TLR2-transfected cells presented an inflammatory response, according to the pathogenesis of acne, when stimulated by *P. acnes*, showing that this receptor is important to mediate the effects of the bacterium.

To confirm if TLR2 mediates *P. acnes* effect, we analyzed the immunomodulatory properties of MSCs from KOTLR2 mice in a TBI model. *P. acnes*-KOTLR2-MSC transplantation resulted in a significant decrease in IL-10 expression compared to control (saline) KOTLR2-MSCs. We also observed that TLR2 is probably not the only mechanism by which *P. acnes* acts since the expression of pro-inflammatory cytokines (IL-6 and TNF-α) remained decreased after *P. acnes*-KOTLR2-MSC transplantation. Pevsner-Fischer et al. (2007), demonstrated that TLR ligands were able to increase MSC proliferation and keep them in an undifferentiated stage. In such study, TLR2 (Pam3Cys) and TLR4 (LPS) agonists were shown do increase de inflammatory environment and, through activation of NF-kB signaling pathway, inhibited MSC differentiation into adipocytes, osteoblasts, chondrocytes and myocytes. However, the same work also demonstrated that MSC from MyD88 deficient animals secreted IL-6 in response to TLR agonist, and no longer via TLR2 or TLR4 (neither by MyD88 independent activation of TLR4), and that these cells also lack the ability to differentiate into osteocytes or chondrocytes. Thus, this same group affirms that TLR signaling must be necessary for acquisition of multipotency by MSCs. In addition, another group demonstrated that TLR4 agonist (LPS), by inducing a highly inflammatory environment, inhibited chondrogenesis by human MSCs by the presence of IL-6, rather than by LPS activation itself (Ruhl et al., 2018). Activation of signaling pathway JAK-STAT by IL-6 down-regulates the expression of cartilage-specific matrix genes, accompanied by the reduction of SOX9, the main regulator of chondrogenesis (Legendre et al., 2003). Further investigation on this is necessary, but it would be possible to infer that while the presence of IL-6 would be unfavorable to differentiation of these cells, important for regeneration, the decrease of IL-6 demonstrated here, together with a less inflammatory environment, would not be impairing the differentiation of these cells. In addition, the MSC origin influences the TLR profile as well as its functional properties (Raicevic et al., 2011). TLR2 and TLR4 agonists, for example, did not influence adipogenic differentiation, and increased the osteogenic and chondrogenic differentiation of human MSCs obtained from umbilical cord (Kim et al., 2010).

As TLR2 is one way by which *P. acnes* modulates MSCs, we could explain its adjuvant effect on MSC absolute number, proliferation and MSC subpopulations.

Taken together it is clear that *P. acnes* enhances the MSC immunomodulatory properties including the identification and function of their subpopulations as well as their proliferative capacity. The soluble PS is an important component that mediates most of the *P. acnes* effects on MSCs. Furthermore, the involvement of TLR2 in the modulatory effects of *P. acnes* was once again evident. These findings show that heat-killed *P. acnes* could be used as an important biologic stimulus to improve MSCs. Freshly sorted MSCs could also be an alternative to the extraction of cells followed by culture since this population possesses modulatory properties. The use of these specific MSC subpopulations in transplantation still depends on further experimental studies.

## Author Contributions

GS, MP and IL-M conceived and designed the experiments. GS, MI, DT, LG and AS performed the experiments, analyzed the data and prepared the figures. GS and IL-M wrote the manuscript. IL-M and MP performed the final review of the article. All authors read and approved the final article.

## Conflict of Interest Statement

The authors declare that the research was conducted in the absence of any commercial or financial relationships that could be construed as a potential conflict of interest.
