# Supplementary material for: Improvement of Mesenchymal Stem Cell Immunomodulatory Properties by Heat-Killed Propionibacterium acnes via TLR2
Source: Front Mol Neurosci. 2019 Jan 10;11:489. doi: 10.3389/fnmol.2018.00489 (PMC6336115; doi:10.3389/fnmol.2018.00489)
Supplement: Supplementary file 1 [file Data_Sheet_1.PDF]

*Supplementary Material*

# IMPROVEMENT OF MESENCHYMAL STEM CELL IMMUNOMODULATORY PROPERTIES BY HEAT-KILLED *PROPIONIBACTERIUM ACNES* VIA TLR2

**Gabriela da Paz Silveira[1], Mayari Eika Ishimura[1], Daniela Teixeira[1], Layla Tesla Galindo[2], Agnes Araujo Sardinha[2], Marimelia Porcionatto[2], Ieda Maria Longo-Maugéri[1*]**

*** Correspondence:** Corresponding Author: Ieda Maria Longo-Maugéri, imaugeri@unifesp.br

## 1 Supplementary Data

| Gene | | Primer Sequences | (pb) |
|---|---|---|---|
| *IL-6* | *Sense* | 5´- TACCACTTCACAAGTCGGAGGC -3´ | 116 |
| | *Antisense* | 5´- CTGCAAGTGCATCATCGTTGTTC -3´ | |
| *TNF-α* | *Sense* | 5´- CTATGTCTCAGCCTCTTCTCATTC -3´ | 114 |
| | *Antisense* | 5´- GAGGCCATTTGGGAACTTCT -3´ | |
| *IL-10* | *Sense* | 5´- CGGGAAGACAATAACTGCACCC -3´ | 130 |
| | *Antisense* | 5´- CGGTTAGCAGTATGTTGTCCAGC -3´ | |
| *IL-4* | *Sense* | 5´- CTCTAGTGTTCTCATGGAGCTG -3´ | 98 |
| | *Antisense* | 5´- GTGATGTGGACTTGGACTCAT -3´ | |
| *TGF-β* | *Sense* | 5´- ACCAACTATTGCTTCAGCTTCAGCTCCAC-3´ | 100 |
| | *Antisense* | 5´- GATCCACTTCCAACCCAGGTC -3´ | |
| *HPRT* | *Sense* | 5´- CTCATGGACTGATTATGGACAGGAC -3´ | 123 |
| | *Antisense* | 5´- GCAGGTCAGCAAAGAACTTATAGCC -3´ | |
| *GAPDH* | *Sense* | 5´- GACAACTTTGGCATTGTG -3´ | 133 |
| | *Antisense* | 5´- ATGCAGGGATGATGTTCTG -3´ | |

**Supplementary Table 1. Sequences of primers used in real time reverse transcription polymerase chain reaction (qPCR) to quantify cytokine expression**

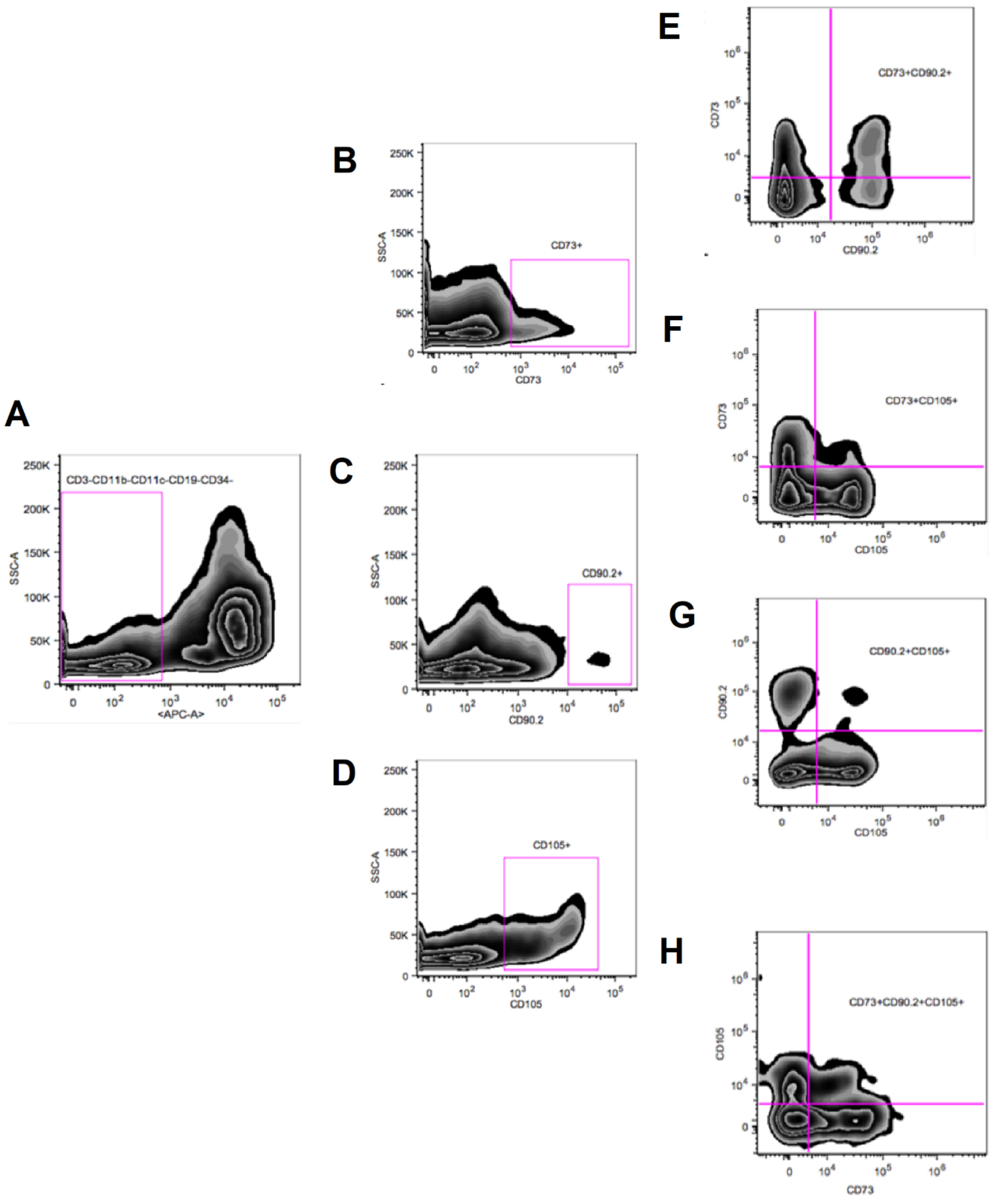


**Supplementary Figure 1. Gate strategy to analyze subpopulations of MSCs freshly isolated from blood and BM.** Mononuclear cells were stained with monoclonal antibodies for CD3, CD11b, CD11c, CD19 and CD34. Cells negative for those markers were sorted by FACS (A). Then, cells were labeled for CD73, CD90.2 and CD105 to characterize and quantify $CD73^+$ (B), $CD90.2^+$ (C), $CD105^+$ (D), $CD73^+CD90.2^+$ (E), $CD73^+CD105^+$ (F), $CD90.2^+CD105^+$ (G) and $CD73^+CD90.2^+CD105^+$ (H) MSC subpopulations. Abbreviations: APC, fluorochrome allophycocyanin; BM, bone marrow; SSC-A, side scatter-area.

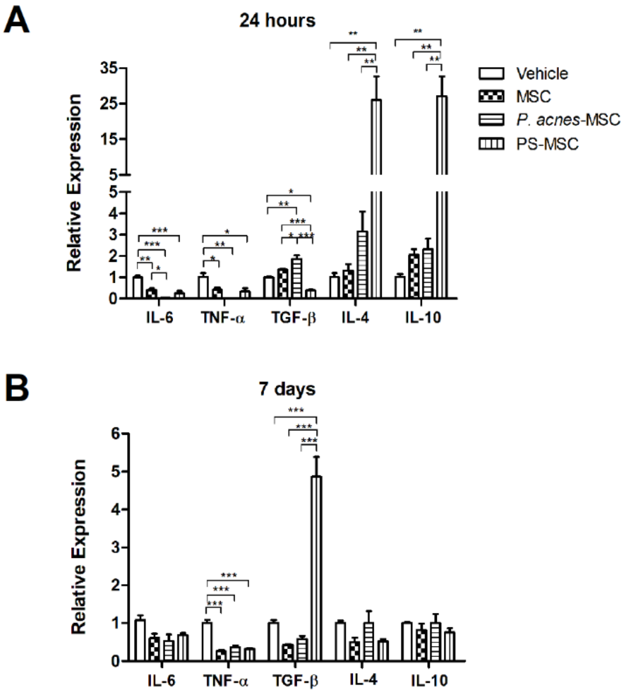


**Supplementary Figure 2. *P. acnes* and PS enhance MSC immunomodulatory properties**. (A) Twenty-four hours and (B) 7 days after TBI and MSC transplantation, RNA was extracted from motor cortex. qPCR was performed, and cytokine expression was calculated relative to *GAPDH* expression. Levels of the pro-inflammatory cytokines IL-6 at 24 hours ($F_{(3,8)}$=29.00, $p$=0,0001) and TNF-α at 24 hours ($F_{(3,8)}$=11.22, $p$=0,0031) and 7 days ($F_{(3,8)}$=44.87, $p$<0,0001) after TBI were significantly decreased in all groups compared with the control group (vehicle). Only *P. acnes*-MSCs reduced IL-6 expression compared with that in the MSC group. Levels of the anti-inflammatory cytokine IL-4 at 24 hours ($F_{(3,8)}$=13.46, $p$=0,0017) were increased in the *P. acnes*-MSC and PS-MSC groups compared with the control and MSC groups. At 7 days, IL-4 expression was reduced in the MSC and PS-MSC groups compared with the control group ($F_{(3,8)}$=2.64, $p$=0,1208). IL-10 expression was increased at 24 hours ($F_{(3,8)}$=20.09, $p$=0,0004) in the *P. acnes*-MSC group compared with the control group. IL-10 expression was also increased after PS-MSC treatment compared with that in the control and MSC groups. TGF-β levels were increased at 24 hours ($F_{(3,8)}$=40.31, $p$<0,0001) in the *P. acnes*-MSC and MSC groups compared with the control group and at 7 days ($F_{(3,8)}$=61.55, $p$<0,0001) in the PS-MSC group compared with the control and MSC groups. Data are expressed as the mean (using the $2^{-\Delta\Delta Ct}$ method) ± SEM of 3 mice per group, from a representative experiment of three independent experiments. *$p$<0.05, **$p$<0.001 and ***$p$<0,0001, determined by one-way ANOVA followed by Tukey's post-test. Abbreviations: qPCR, real time polymerase chain reaction; TBI, traumatic brain injury.

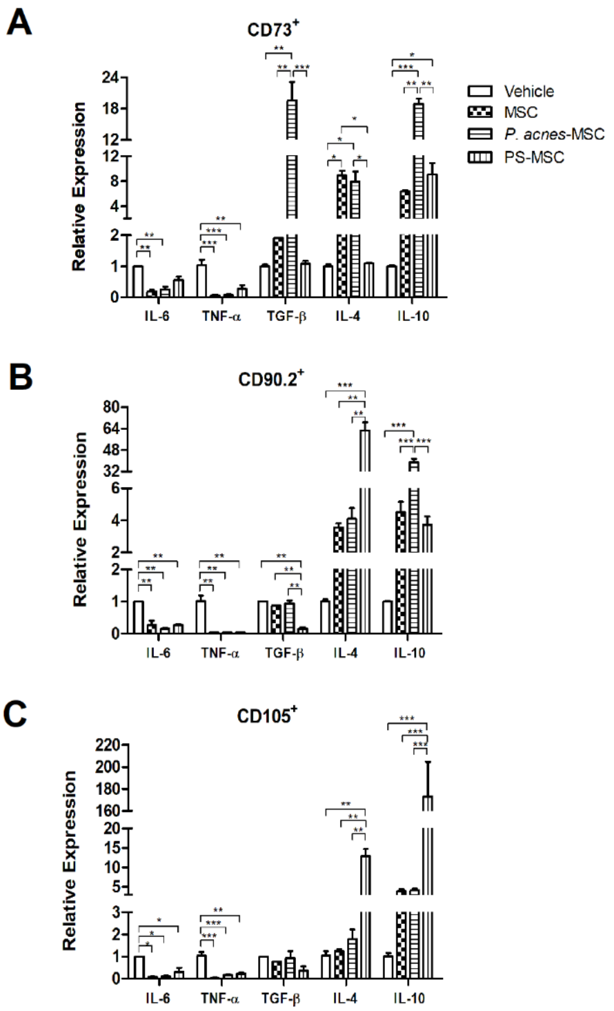


**Supplementary Figure 3.** ***P. acnes*** **and PS enhance MSC subpopulation immunomodulatory properties**. Twenty-four hours after TBI and MSC subpopulation transplantation (A, CD73$^+$ MSC; B, CD90.2$^+$ MSC; and C, CD105$^+$ MSC), RNA was extracted from motor cortex. qPCR was performed, and cytokine expression was calculated relative to *GAPDH* expression. Compared with that in the control group (vehicle), expression of the pro-inflammatory cytokines IL-6 and TNF-α after TBI was significantly decreased in all groups after transplantation with CD73$^+$ ($F_{(3,4)}$=22.33, $p$=0,0058; $F_{(3,8)}$=19.24, $p$=0,0005), CD90.2$^+$ ($F_{(3,4)}$=32.40, $p$=0,0029; $F_{(3,4)}$=33.22, $p$=0,0028)and CD105$^+$ ($F_{(3,4)}$=21.75, $p$=0,0061; $F_{(3,8)}$=23.83, $p$=0,0002) MSCs. Compared to that in the control and MSC groups, TGF-β expression was enhanced only in the *P. acnes*-MSC-CD73$^+$ group ($F_{(3,4)}$=26.50, $p$=0,0042). Expression of the anti-inflammatory cytokine IL-4 was increased in the *P. acnes*-MSC-CD73$^+$ ($F_{(3,4)}$=23.73, $p$=0,0052), *P. acnes*-MSC-CD90.2$^+$ ($F_{(3,4)}$=95.01, $p$=0,0004), PS-MSC-CD90.2$^+$, and PS-MSC-CD105$^+$ ($F_{(3,4)}$=41.25, $p$=0,0018) groups compared to that in the control group and in the PS-MSC-CD90.2$^+$ and PS-MSC-CD105$^+$ groups compared to that in the MSC group. IL-10 expression was increased in all groups after transplantation with CD73$^+$ ($F_{(3,4)}$=53.04, $p$=0,0011), CD90.2$^+$ ($F_{(3,4)}$=155.6, $p$=0,0001) and CD105$^+$ ($F_{(3,8)}$=29.15, $p$=0,0001) MSCs compared with that in the control group (vehicle). Compared to MSC transplantation, only *P. acnes*-MSC-CD73$^+$, *P. acnes*-MSC-CD90.2$^+$, and PS-MSC-CD105$^+$ transplantation enhanced IL-10 expression. Data are expressed as the mean (using the $2^{-\Delta\Delta Ct}$ method) ± SEM of 3 mice per group, from a representative experiment

of three independent experiments. *$p<0.05$, **$p<0.001$ and ***$p<0.0001$, determined by one-way ANOVA followed by Tukey’s post-test. Abbreviations: qPCR, real time polymerase chain reaction; TBI, traumatic brain injury.

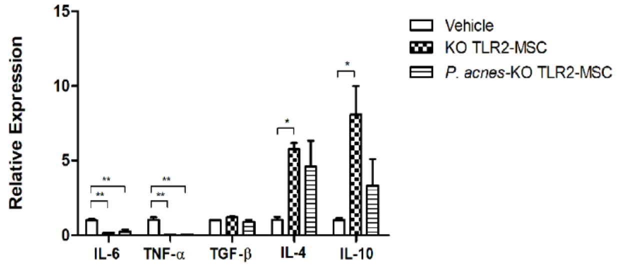


**Supplementary Figure 4. TLR2 as an important mechanism of the effects of *P. acnes* on MSCs.** Twenty-four hours after TBI and TLR2 knock out MSC transplantation in a wild type mouse, RNA was extracted from motor cortex. qPCR was performed, and cytokine expression was calculated relative to *GAPDH* expression. Expression of the pro-inflammatory cytokines IL-6 ($F_{(2,6)}$=20.63, $p$=0,0020) and TNF-α ($F_{(2,6)}$=32.62, $p$=0,0006) was significantly decreased in all groups compared to that in the control group (vehicle). No difference in TGF-β expression was observed ($F_{(2,6)}$=1.95, $p$=0,2221). IL-4 expression was increased after KOTLR2-MSC and *P. acnes*-KOTLR2-MSC treatment ($F_{(2,6)}$=5.76, $p$=0,0402). IL-10 expression was elevated only after KOTLR2-MSC transplantation ($F_{(2,6)}$=5.74, $p$=0,0404). Data are expressed as the mean (using the $2^{-\Delta\Delta Ct}$ method) ± SEM of 3 mice per group, from a representative experiment of three independent experiments. *p<0.05, and **p<0.001, determined by one-way ANOVA followed by Tukey's post-test. Abbreviations: KO, knock out; qPCR, real time polymerase chain reaction; TBI, traumatic brain injury.
